# Molecular Signatures of the Insulin-Like Growth Factor 1-Mediated Epithelial-Mesenchymal Transition in Breast, Lung and Gastric Cancers

**DOI:** 10.3390/ijms19082411

**Published:** 2018-08-15

**Authors:** Armando Cevenini, Stefania Orrù, Annamaria Mancini, Andreina Alfieri, Pasqualina Buono, Esther Imperlini

**Affiliations:** 1 Dipartimento di Medicina Molecolare e Biotecnologie Mediche, Università degli Studi di Napoli “Federico II”, Via S. Pansini 5, 80131 Napoli, Italy; armando.cevenini@unina.it; 2 CEINGE-Biotecnologie Avanzate S.c.a r.l., Via G. Salvatore 486, 80145 Napoli, Italy; annamaria.mancini@uniparthenope.it (A.M.); andreina.alfieri@uniparthenope.it (A.A.); 3 Dipartimento di Scienze Motorie e del Benessere, Università degli Studi di Napoli “Parthenope”, Via Medina 40, 80133 Napoli, Italy; orru@uniparthenope.it (S.O.); pasqualina.buono@uniparthenope.it (P.B.); 4 IRCCS SDN, Via Francesco Crispi 8, 80121 Napoli, Italy

**Keywords:** insulin-like growth factor 1 (IGF-1), IGF-1 receptor (IGF-1R), epithelial-mesenchymal transition (EMT), cancer, metastasis, PI3K/AKT pathway, RAS/MEK/ERK pathway

## Abstract

The insulin-like growth factor (IGF) system, which is constituted by the IGF-1 and IGF-2 peptide hormones, their corresponding receptors and several IGF binding proteins, is involved in physiological and pathophysiological processes. The IGF system promotes cancer proliferation/survival and its signaling induces the epithelial-mesenchymal transition (EMT) phenotype, which contributes to the migration, invasiveness, and metastasis of epithelial tumors. These cancers share two major IGF-1R signaling transduction pathways, PI3K/AKT and RAS/MEK/ERK. However, as far as we could review at this time, each type of cancer cell undergoes EMT through tumor-specific routes. Here, we review the tumor-specific molecular signatures of IGF-1-mediated EMT in breast, lung, and gastric cancers.

## 1. Introduction

The secreted protein hormones insulin-like growth factors (IGFs) are key modulators of several physiological processes and participate in various pathophysiological events [1,2,3,4,5]. Although the IGF system is neither genotoxic nor transforming, its enhanced activation promotes cancer proliferation and survival. The epithelial-mesenchymal transition (EMT) plays a crucial role in migration and invasiveness of neoplastic cells thereby leading to metastases [6]. Experimental evidence obtained in the last two decades suggests that IGF-1 is able to induce EMT in cancer [7]. Here, we review the state-of-the-art knowledge about the IGF-1-mediated EMT activation with particular focus on the tumor-specific molecular signatures in breast, lung and gastric cancers.

## 2. The IGF System

The whole IGF system is characterized by two secreted hormones (IGF-1 and IGF-2), two receptors (IGF-1R and IGF-2R) and several binding proteins (IGFBP1-7) [8,9]. Generally, IGFBPs modulate the bioavailability of both IGFs by handling their release, transport and degradation [10], and IGFBP3 carries 75% or more of the two circulating hormones [11]. IGF-1 and IGF-2 share sequence homology with insulin [12] and are mainly produced by the liver [13]. Also extra-hepatic organs secrete these growth factors, thereby enabling endocrine and paracrine/autocrine signaling [14]. At the physiological level, hepatic secretion of IGF-1 is dependent by growth hormone (GH) action [15], and circulating IGF-1 levels are linked to anabolic functions and growth-promoting processes because the GH/IGF axis favors the development and differentiation of several tissues [16,17]. IGF-1 levels can vary among healthy individuals depending on sex, age, and lifestyle factors [18], but their interpersonal variability is considered a cancer risk determinant [3]. Nutrition is one of the major regulators of circulating IGF-1 levels [19,20]. In fact, fasting reduces serum IGF-1 levels, whereas long-term calorie restriction is less effective, particularly if coupled to a high protein intake [21,22,23]. Also exercise affects IGF-1-dependent signaling by reducing circulating hormone levels and/or by increasing the expression of specific IGFBPs [24,25]. The ability of IGFBPs to limit the amount of free IGF-1 in blood partly explains the molecular mechanisms underlying cancer prevention by exercise [24].

The complexity of the IGF system is also linked to IGF-1 mRNA alternative splicing events, which generate three pro-forms in humans: proIGF-1Ea, proIGF-1Eb, and proIGF-1Ec. Intracellular IGF-1 seems to be mainly expressed as proIGF-1 that is extracellularly converted into mature IGF-1 [26,27]. Although IGF-1’s biological activity is mainly exerted by the mature peptide, recent evidence suggests that the various prohormones exert functional roles [28]. These prohormones share the same mature IGF-1 sequence, but have different C-terminals, also known as “E-peptides”. The expression of proIGFs differs between normal and tumor tissues/cells [29]. Moreover, proIGFs differ in posttranscriptional modifications: proIGF-1Ea, the most abundant prohormone, has a highly conserved N-glycosylation site (N92) in its E-peptide, whereas proIGF-1Eb and proIGF-1Ec lack N-glycosylation sites in their E-domains [29]. Interestingly, it was recently demonstrated that E-peptides play a key role in controlling the subcellular localization and cellular trafficking of proIGFs and, as a consequence, in controlling the regulation of mature IGF-1 production, secretion and bioavailability [26,28].

Unlike IGF-1, IGF-2 is an epigenetically regulated gene mainly involved in fetal development [12] and expressed by both alleles only in early tumor cells [30], which suggests that IGF-2 plays a critical role in oncogenesis [31]. IGF signaling is mainly mediated via activation of the receptor tyrosine kinases (RTKs) IGF-1R and IGF-2R [1,4,17], both of which being able to complex with insulin receptor (IR) [13]. IGF-1, IGF-2 and insulin bind their corresponding receptors with high affinity, but hybrid ligand/receptor complexes also occur [13]. IGF-1R is coupled to several downstream pathways, namely, the PI3K/AKT, RAS/RAF/MAPK, and STAT cascades, after ligand binding and phosphorylation of docking sites [13]. Consequently, IGF-1R triggers growth-promoting and anti-apoptotic signals. This transmembrane receptor is vital for cells and tissues; in fact, IGF-1R(−/−) mice are smaller than their normal counterparts and die soon after birth from respiratory failure [32]. However, murine embryos lacking IGF-1R do not develop cancer when exposed to oncogenes [33]. Although ligated as well as unligated IGF-1R is neither genotoxic nor transforming [34], its expression increases at mRNA and protein levels [35] in pediatric, brain, renal and hematological tumors, and in transformed cells [1,3]. In these contexts, IGF-1R signaling contributes to the mitogenic and anti-apoptotic cascades and to migration, metastasis and angiogenesis [36]. In fact, after an IGF-1-independent oncogenic event, survival of the newly transformed cells depends largely on the IGF system.

The evaluation of IGF-1R in epithelial tumors in adults is complex due to the interplay between IGF-1R and such other cellular factors [37,38,39,40,41] as stimulatory and/or inhibitory transcriptional regulators [42]. For example, androgens and estrogens can stimulate *IGF-1R* gene expression in steroid hormone-dependent tumors such as breast, prostate, and even bone [43,44], whereas IGF-1R levels may be reduced in steroid hormone-independent tumors [36]. However, conflicting data have been reported regarding these issues [45]. IGF-1R expression levels are rarely related to a mutation in the corresponding gene; the few known cases describe heterozygote forms associated to growth retardation and not to neoplasia [46,47,48]. It is conceivable that “loss-of-function” and “gain-of-function” mutation theory can clarify the role played by the *IGF-1R* gene in cancer [36]. In the former case, the mutated tumor suppressors p53, BRCA1, and VHL [49,50,51] are not able to inhibit downstream targets, including *IGF-1R*. In the latter case, the transactivation of the IGF-1R promoter would be enhanced [52]. In fact, enhanced IGF-1R transactivation by oncogenic fusion proteins has been observed in pediatric and adolescent tumors [53,54,55], and recently, also in adult epithelial tumors [56]. An intact and activated IGF-1R signaling network is required to commit a cell to neoplastic transformation [36,52]. In fact, according to the adoption phenomenon, oncogenes exert their action through IGF-1R signaling that mediates the oncogene-directed differentiation events [36,52]. This mechanism of action is exerted by pp60^src^ [57], c-myb [58,59] and hepatitis B virus X oncogenes [60]. Jones et al. suggested that in transformed cells overexpressing IGF-1R, the latter’s proliferative/survival signaling can be ligand-independent above a certain expression level, whereas in normal cells in which the receptor is under expressed, IGF stimulation triggers a robust response [61]. Such ligand-independent/dependent signaling may play an instrumental role when IGF-1 is used at supraphysiological doses as a performance-enhancing drug [15,62,63]. In fact, IGF-1 is included in the World Anti-Doping Agency list of banned peptide hormones. In this context, there is no convincing evidence that the GH/IGF axis improves sport performance in terms of increased muscle mass and strength, enhanced muscle protein synthesis and fatty acid availability, and reduced glycogen consumption [64,65,66]. Conversely, supraphysiological doses of IGF-1, alone or combined with other doping agents, result in a molecular signature resembling a system prone to carcinogenesis in human peripheral mononuclear cells expressing physiological levels of IGF-1R [67,68,69]. Similarly, the IGF-1/IGF-1R axis can stimulate other concurrent oncogenic mechanisms in tumor cells, such as androgen receptor signaling in prostate [70,71] and in colon cancer cells [72,73].

Another level of IGF-1R expression regulation occurs at transcriptional level by means of microRNAs (miRs) [74]. Jiang et al. suggested that miR-7 plays a role in the modulation of IGF-1R expression in tongue squamous carcinoma cells [75], whereas McKinsey et al. proposed a novel complex mechanism in Ewing sarcoma (EWS) in which a set of miRs able to regulate negatively pro-oncogenic IGF-1R pathway components was repressed by the EWS-Fli1 oncogene [76]. 

Within the complex interplay between the IGF system and cancer, IGF-1R signaling is involved in the metabolic shift of transformed cells from an aerobic to an anaerobic production of ATP, known as the “Warburg effect” [77]. In particular, the PI3K pathway in cancer cells triggers not only anti-apoptotic and mitogenic signals but it also affects cellular metabolism by stimulating the glycolytic pathway [78] as well as lipid and protein synthesis [79].

## 3. The IGF System and Breast, Lung, and Gastric Cancers

The IGF system contributes to the progression and evolution of several epithelial cancers and its components often acquire a prognostic significance. The IGF-1/IGF-1R signaling axis is involved in breast carcinogenesis and development [80]. Breast cancer (BC) is the most frequently diagnosed malignant tumor in women and its 5-year survival rate is 85% or higher [81,82,83]. However, at least 20% of all patients develop metastatic BC with an average survival of less than two years [83]. As a first-line therapy of metastatic BC, a cisplatin plus gemcitabine regimen are more effective than paclitaxel plus gemcitabine [84]. However, current chemotherapies are often associated with drug resistance, reduced potency, and relevant side effects [85]. To address these issues, attempts are being made to unravel the molecular targets/signaling involved in the altered biological processes. In fact, IGF-1/IGF-2 overexpression has been correlated with BC development, aggressive phenotypes, and with BC cell survival and proliferation [80,86,87]. Indeed, increased serum levels of IGF-1 have been associated with a high risk of BC [42,88,89,90]. A growing body of evidence indicates that the IGF-1/IGF-1R signaling pathway is a key player in BC cell therapy resistance and cancer recurrence [91,92,93]. Hence, most clinical studies have focused on this pathway both as a target for BC treatment, and for prevention [93]. In particular, circulating IGF-1 and/or IGF-1R expression levels could serve as predictive biomarkers to predict which patients will respond to IGF-1R-targeted therapies [94,95]. However, some early-phases clinical studies reported that changes in the IGF-1/IGF-1R axis may be a necessary but not sufficient condition to define the patients’ responsiveness [96,97,98].

The potential role of IGF-1/IGF-1R signaling in cancer progression has been investigated also in lung cancer (LC). Lung cancer is the most common cause of cancer deaths worldwide, with a slightly lower incidence in women than in men, and more than 75% of cases are non-small cell lung cancers (NSCLC) [99]. Histologic subtypes and clinical stages seem to influence IGF-1R expression and serum IGF-1/IGFBP3 levels. In fact, Agullo-Ortuno et al. reported differential expression of IGF-1R across histologic subtypes with reduced levels in squamous cell tumors [100]. On the contrary, IGF-1R is overexpressed in small cell lung cancer (SCLC) and its inhibition affects cancer cell growth [101]. In patients with both NSCLC histology and NSCLC metastatic stage, serum IGF-1/IGFBP3 levels are significantly higher than those measured in subjects with SCLC histology and non-metastatic stage of NSCLC [102]. However, neither circulating IGF-1 nor IGFBP3 have been associated with the clinical outcome of LC [102]. The chemotherapy protocols used in LC patients did not affect these circulating components of the IGF system. In fact, after chemotherapy, NSCLC and SCLC patients continued to have higher IGF-1 levels and lower IGFBP concentrations *versus* controls [103]. Notably, IGFBP3 expression is reduced in cisplatin-resistant LC cells and the in vitro IGFBP3 overexpression induces apoptosis and improves drug response [104]. Consequently, IGFBP3 may be a predictive marker of LC patients responsive to IGF-1-targeted therapy.

Genetic variants of IGFBP3 were found to correlate significantly with the survival of patients affected by advanced gastric cancer (GC), which is the most frequent cause of cancer-related mortality [105,106,107,108,109]. Gastric cancer is often diagnosed in an advanced stage when the patient has widespread metastases, and its 5-year survival rate is 28% or less [105,110,111]. So far, there are no specific biomarkers for an early diagnosis and metastasis prediction [105,109]. Conventional anticancer and targeted therapies have slightly improved prognosis, but not in patients with metastasis [105,108]. Stage IV patients are usually treated with cisplatin/oxaliplatin and fluoropyrimidine chemotherapy, combined with trastuzumab in EGFR2-positive patients [9,110]. Moreover, in patients treated with oxaliplatin, 5-fluorouracil and leucovorin, the presence of single nucleotide polymorphisms in *IGF-1R* and *IGF-1* are significantly associated with treatment response [112]. The levels of co-expression of IGF-1R and multidrug resistance-associated protein-1 (MRP-1) in the tumor may predict the effect of chemotherapy [113]. Indeed, IGF-1R expression is an independent predictor of survival in patients and has been correlated with a worse prognosis and lymph node metastasis. In addition, IGFBP7 levels were found to correlate positively with tumor stage, invasiveness and metastatic capability [9].

## 4. Epithelial-Mesenchymal Transition (EMT)

Most epithelial cancer cells are characterized by intrinsic plasticity that, in specific conditions, give rise to the EMT. This is a multistep process by which cells modify their own morphological and functional features and acquire mesenchymal characteristics [6,114]. Mesenchymal cancer cells become invasive thanks to their enhanced motility, and can pass through surrounding tissues [115,116]. The EMT is a key step in the process that characterizes the dismal prognosis of most epithelial cancers and carcinomas, promoting the survival, self-renewal and metastatic potential of cancer cell subtypes, generally referred to as cancer stem cells (CSCs) [117,118,119,120]. Indeed, the EMT process is triggered by the interaction between the inherent potential of cancer epithelial cells to acquire the mesenchymal phenotype and environmental and/or autologous stimuli and growth factors [6,120,121]. A molecular hallmark of the EMT process is the reduction, delocalization, and degradation of E-cadherin, which results in the dissolution of adherent cell junctions. Moreover, β-catenin, no longer able to bind E-cadherin, can exert its transcriptional activity in crosstalk with WNT signaling [120,121,122,123,124]. While the expression of specific membrane proteins is downregulated during the EMT, alternative gene expression programs are activated to promote mesenchymal adhesion [6,120,121]. In detail, the reduction of E-cadherin expression is counterbalanced by N-cadherin upregulation that mediates feeble cell interactions that favor migration capability [6,125]. N-cadherin interacts with the neural cell adhesion molecule (NCAM) that modulates the activity of specific RTK pathways and hence promotes cell migration [6]. Cytoskeletal intermediate filaments also change their composition because cytokeratin expression is impaired during EMT in favor of vimentin upregulation. This mechanism influences motor protein functions and the trafficking of organelles and membrane proteins, thus favoring the mesenchymal capability of migration and invasion [6,126,127]. Cells that have undergone the EMT loose interaction with the basement membrane and acquire the ability to interact with different extracellular matrices (ECMs) by regulating the expression of specific integrins and also matrix metalloproteinases (e.g., MMP2, MMP9) [6,122,128,129,130,131,132]. 

The molecular mechanisms controlling the EMT, namely, the master regulators of the transition are subdivided into two groups: (i) transcription factors and (ii) signaling pathways.

### 4.1. Transcription Factors

During the EMT, a complex switch in gene expression program occurs via transcription factors belonging mainly to three protein families: SNAIL, helix-loop-helix (bHLH), including TWIST, and zinc finger E-box binding homeobox (ZEB) proteins [6,120,121]. These factors often influence the expression of each other, frequently converge in the regulation of common target genes and in general upregulate mesenchymal genes and repress epithelial genes [6,120,121,133,134]. SNAIL1 and SNAIL2, also known as SNAIL and SLUG, respectively, activate the EMT program [6,133,135,136,137,138,139,140,141,142,143,144]. Most EMT-promoting signaling pathways (TGF-β, WNT, Notch, and RTKs) activate SNAIL1 expression, and control its localization and stability [120,121,133]. GSK-3β phosphorylates specific serine residues of SNAIL1, thus inducing its nuclear export, ubiquitination, and consequent degradation. The WNT, PI3K/AKT, Notch and NF-κB pathways enhance SNAIL1 stability by inhibiting its GSK-3β-mediated phosphorylation or simply by preventing GSK-3β binding [6]. The transcription factors bHLHs include some regulators of EMT program, namely, E12, E47, TWIST1, and TWIST2 that repress epithelial phenotype genes (e.g., E-cadherin) and promote the expression of mesenchymal genes (e.g., N-cadherin) via SNAIL-independent routes [120,121,133,134,136]. Many signaling pathways upregulate TWISTs during cancer development and tumorigenesis [120,121,133,136]. Furthermore, microenvironmental conditions, e.g., hypoxia, can increase TWIST expression and favor EMT by inducing hypoxia-inducible factor 1α (HIF1α) [6,120,145]. Similar to SNAILs, also TWIST1 stability is regulated by MAPKs through phosphorylation that impedes ubiquitination and degradation of TWIST1 [146].

The two ZEB transcription factors, namely ZEB1 and ZEB2, can act both as transcriptional repressors and activators [136,147]. ZEBs downregulate genes encoding proteins involved in epithelial cell-cell junctions and in the maintenance of apical-basal polarity, and upregulate mesenchymal genes [133,136]. In most cases, increased ZEB expression can be a consequence of SNAIL activation. In fact, as an example, the *ZEB1* gene is a direct target of SNAIL1 transactivity, whereas TWIST1 can improve this effect by cooperating with SNAIL1 [148]. The expression of ZEBs is promoted by the TGF-β, WNT and RAS pathways [136], while it is inhibited by specific miRs, namely, miR-200, miR-205, and miR-192 families [6,121].

### 4.2. Signaling Pathways

Among the proteins of the TGF-β superfamily, TGF-β1 and TGF-β2 are the most powerful signals able to trigger the EMT program. In particular, TGF-β1 activity induces the EMT in diverse carcinomas, and leads to cancer cell migration and metastasis [115,149,150]. SMAD proteins are intracellular effectors of the TGF-β signaling that control EMT gene expression both directly and by upregulating the expression and activity of EMT transcription factors [6,121,151,152]. TGF-β-activated SMADs can induce SNAIL expression and cooperate with the latter to downregulate E-cadherin [153]. TGF-β can also promote ZEB1 expression, which is also controlled by MAPK signaling [154]. The interaction of SMAD with ZEB1 and ZEB2 enhances TWIST expression by downregulating the inhibitor of DNA binding 1 (ID1) [155], and directly inducing the expression of genes encoding mesenchymal phenotype proteins e.g., fibronectin, vimentin and collagen αI [156,157]. 

TGF-β induces signaling through the RHO-like GTPases, PI3K and MAPK pathways, all of which contribute to the EMT [121,136,158,159,160,161]. Activation of RHO-like GTPases drives cytoskeletal reorganization and lamellipodia and filopodia formation [162]. TGF-β also activates PI3K/AKT, which results in activation of mammalian TOR complex 1 (mTORC1) and mTORC2 [120,121,163,164,165]. Indeed, mTORC1 mediates the processes leading to increased cell motility and invasion [164] and mTORC2 is crucial for acquisition of the mesenchymal phenotype [163]. AKT also sustains SNAIL1 expression and participates in E-cadherin repression and MMP upregulation [163,166]. It also phosphorylates GSK-3β, thereby inhibiting the activity of the latter and consequently enhancing SNAIL1 stability [6]. TGF-β-dependent ERK/MAPK signaling is mediated by the adaptor protein SRC homology 2 domain-containing-transforming A (SHCA), which binds growth factor receptor-bound protein 2 (GRB2) and the son of seven less (SOS) genes, thereby triggering the RAS/RAF/MEK/ERK cascade [167].

Growth factors recognized by RTKs can mediate an induction of EMT mainly via PI3K/AKT and MAPK/ERK [6]. As in the case of TGF-β, also in RTK-signaling, the AKT and mTORC2 pathway is crucial for EMT activation [6,168]. Mutations in the genes encoding RAS or RAF also promote RTK-mediated EMT in cancer cells. The RAS pathway induces SNAIL1 and SNAIL2 expression and promotes activation of the RHO-GTPases thereby favoring migratory and invasive properties in cancer cells [169]. Indeed, RTK signaling promotes TGF-β1 expression, thus potentiating its signaling [6]. IGF-1 induces the EMT in most cancer cells, whereas IGF-1R activation leads to downregulation of E-cadherin and upregulation of N-cadherin, vimentin, and fibronectin [170]. Indeed, IGF-1R complexes with E-cadherin and αv integrin are destabilized by IGF-1 thereby promoting cell motility [171]. In some epithelial cells, IGF-1-mediated activation of NF-κB upregulates SNAIL1 [170] while in other cells, the IGF-1-mediated EMT results from the activation of the MAPK/ERK axis with consequent enhancement of ZEB1 expression [172]. Lastly, the PI3K/AKT pathway is essential for IGF-1-induced EMT in all the epithelial cells [173].

## 5. IGF-1 Signaling and EMT Activation in Breast, Lung, and Gastric Cancers

### 5.1. Breast Cancer

Experimental and clinical evidence indicates that IGF-1 induces the EMT phenotype, thereby promoting BC cell growth, survival, migration, invasiveness, metastasis and, eventually, drug-resistance [7,117,174]. In fact, it is now recognized that ERK/MAPK and IRS-1/PI3K/AKT/GSK-3β, the major IGF-1R signaling transduction pathways, are involved in IGF-1-mediated EMT activation in BC. Interestingly, in vitro studies showed that also human proIGFs can promote cell proliferation and migration in different human cell types, both normal and cancer cells including BC [27,29]. In fact, De Santi et al. demonstrated that proIGFs induce BC cell proliferation via IGF-1R activation [27]. ProIGFs activate either the MAPK or the PI3K/AKT arm of IGF-1R downstream signaling in a cell type-dependent manner. Contrary to transgenic mice overexpressing IGF-1Ea, AKT phosphorylation is affected by glycosylated pro-IGF-1Ea in MCF-7 human breast (adenocarcinoma) epithelial cells [27,29]. However, among proIGFs, we cannot exclude the existence of distinct downstream signaling pathways that are divergent from that of mature IGF-1. 

**Figure 1:**
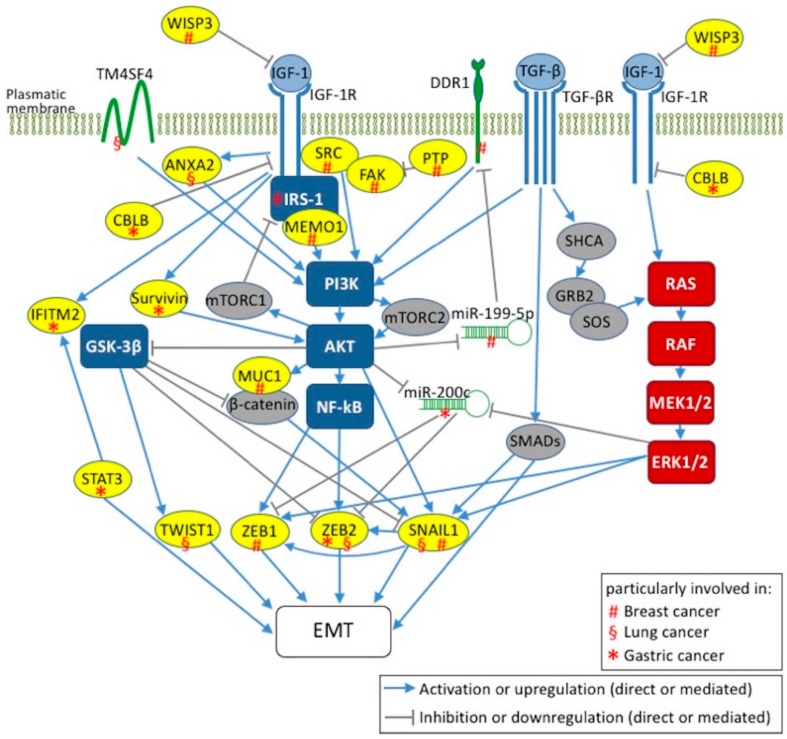
Two major signaling axes in IGF-1-mediated EMTs in human breast, lung, and gastric cancers. IGF-1/IGF-1R signaling axis via IRS-1/PI3K/AKT/GSK-3β/NF-κB and RAS/RAF/MEK/ERK pathways leads to the activation of EMT markers, namely ZEB1/2, SNAIL1 and TWIST1. The interplay between IGF-1 and TGF-β signaling pathways for EMT induction is also indicated. The key mediators involved in EMT induction by IGF-1 are labeled with (#) in breast, (§) in lung cancers and (*) in gastric. WISP3: WNT1 inducible signaling pathway protein 3; TM4SF4: transmembrane 4L six family member 4; ANXA2: annexin A2; CBLB: Cbl proto-oncogene B; IFITM2: interferon induced transmembrane protein 2; STAT3: signal transducer and activator of transcription 3; MUC1: mucin1; DDR1: discoidin domain receptor 1; MEMO1: mediator of ErbB2-driven cell motility 1; PTP: phosphotyrosine phosphatase; FAK: focal adhesion kinase.

Breast cancer-specific routes and specific key mediators are emerging within the canonical IGF-1 signaling transduction pathways that lead to EMT activation (Figure 1). In this context, Kim et al. demonstrated that overexpression of a constitutively active IGF-1R in normal human breast epithelial cells, MCF10A, causes transformation and xenograft growth, which are processes linked to NF-κB-mediated EMT induction via upregulation of SNAIL and downregulation of E-cadherin [170]. In IGF-1-stimulated MCF10A, AKT isoforms play different roles in regulating the EMT process: if AKT1 is downregulated and AKT2 expressed, the stimulated BC cells acquire an EMT phenotype in which ERK/MAPK signaling is activated and cell migration is enhanced [173]. Intriguingly, this phenotype is reversed by AKT2 downregulation, which supports the therapeutic potential of AKT inhibitors in the treatment of specific BCs also coupled with conventional therapies. Notably, overexpression of IGF-1R in MCF-7 together with IGF-1 treatment induces the EMT phenotype in a PI3K–dependent manner [175]. In MCF-7 and highly metastatic MDA-MB-231 human BC cells, stimulation with IGF-1 induces the overexpression of discoidin domain receptor 1 (DDR1), a collagen receptor tyrosine-kinase involved in EMT-dependent cancer progression (Figure 1) [176]. In fact, the cross-talk between IGF-1R and DDR1 is mediated by a PI3K/AKT/miR-199a-5p signaling cascade (Figure 1) [176]. This novel pathway may be manipulated to modulate the IGF-1/IGF-1R axis in BC. In fact, in the experimental model proposed, AKT activation, through the IGF-1 paracrine/autocrine signal, inhibits miR-199a-5p, which in turn induces DDR1 upregulation. Interestingly, DDR1 upregulation and AKT activation were inhibited in BC cells transfected with pre-miR-199a-5p, and as a consequence, cancer cell migration and proliferation were impaired [176]. These findings could open the way to DDR1 inhibitors that can be combined with IGF-1R-targeted therapies for BC treatment. 

Among potential therapeutic targets for BC, Sorokin et al. found that the mediator of ErbB2-driven cell motility 1 (MEMO1) is involved in IGF-1-mediated EMT induction in BC cell lines, mostly in MCF10A cells [177]. Upon binding of MEMO1 to IRS-1, which results in activation of the downstream PI3K/AKT signaling pathway, the EMT is induced via upregulation of SNAIL1 (Figure 1) [177]. It is noteworthy that in normal breast epithelial cells, such as MCF10A, MEMO1, overexpression enhances proliferation and migration, whereas MEMO1 knockdown in highly metastatic MDA-MB-231 cells reverses their invasive phenotype. This suggests that MEMO1 plays a role in BC treatment [177]. Contrary to MEMO1, whose overexpression triggers the EMT in MCF10A cells, the reduction of the matricellular protein WNT1 inducible signaling pathway protein 3 (CCN6/WISP3) activates the IGF-1/IGF-1R axis and induces the EMT via upregulation of ZEB1 (Figure 1) [178]. 

Another potential target for the prevention of BC invasion is mucin 1 (MUC1), which is a transmembrane glycoprotein that acts as a metastasis-promoting oncoprotein. MUC1 is overexpressed in IGF-1 stimulated MCF-7 and MDA-MB-231 cells. MUC1 upregulation via the PI3K/AKT signaling pathway plays a key role in EMT induction that is prevented by MUC1 knockdown in MCF-7 cells (Figure 1) [179]. 

The interplay between the IGF-1 and TGF-β1 signaling pathways is another important feature for EMT induction (Figure 1) [180]. IGF-1 stimulation of MCF-7 cells, through the PI3K and MAPK pathways, leads to the activation of MMPs; this, in turn, activates TGF-β1 with consequent EMT induction after β-catenin nuclear translocation [180]. Stimulation of MCF-7 and BT474 (breast invasive ductal carcinoma) cells with TGF-β induces both the EMT program and nongenomic estrogen receptor-α (ER-α) signaling, which translates into BC progression [181]. Interestingly, the EMT phenotype of these BC cells is characterized by: (i) overexpression of the epidermal growth factor receptor (EGFR) and of IGF-1R which form complex with ER-α; (ii) MAPK activation and, most importantly, (iii) increased resistance to tamoxifen. This reduced sensitivity to anti-estrogen-based therapy is reversed by inhibitors against TGF-β, EGFR, IGF-1R and MEK1/2 [181]. 

Besides the MAPK, PI3K/AKT and NF-κB pathways, also integrins and components of focal adhesion complexes play crucial roles in the TGF-β- and/or IGF-1-induced EMT [175,182]. In fact, IGF-1-induced depolarization of breast epithelial cells involves phosphotyrosine phosphatase activity, which is required for dephosphorylation of focal adhesion kinase (FAK) [175]. It is noteworthy that the IGF-1R-induced EMT features in triple negative breast cancer (TNBC) cell lines are mediated by FAK activation (Figure 1) [183]. In fact, IGF-1R overexpression promotes TNBC migration and invasion and, intriguingly, this phenotype can be abolished by using pharmacological FAK inhibitors [183]. 

### 5.2. Lung Cancer

As in other cancers, activation of ERK1/2, AKT and IKBα/NF-κB is thought to promote carcinogenesis and invasion in NSCLC [184]. NSCLC is also associated to EGFR gene mutations and protein overexpression [185]. EGFR is a transmembrane RTK and tyrosine kinase inhibitors (TKIs), mainly gefitinib and erlotinib, are used in LC therapy. However, EGFR-TKI-treated patients develop drug resistance in less than 1 year [185]. The two mechanisms known to underlie acquired resistance (the secondary EGFR T790M mutation and *c-Met* gene amplification) account for about 60–70% of cases [186,187,188,189,190]. Investigations to identify the causes of the remaining cases focus on mutations in other key genes, impaired signaling and the EMT [191,192,193,194,195,196]. In this context, Zhou et al. support the involvement of the EMT in the drug resistance mediated by IGF-1R in advanced NSCLC cells [186]. In particular, they found that in two NSCLC cell lines (gefitinib-resistant PC9 and erlotinib-resistant H460) IGF-1R and pIGF-1R, but not EGFR, are overexpressed. They also found that exogenous IGF-1 induced IGF-1R activation, enhanced resistance to EGFR-TKIs and, consequently, upregulation of SNAIL, EMT induction by TGF-1β and reduced sensitivity to EGFR-TKIs, whereas E-cadherin overexpression restored this sensitivity by reverting the transition. These findings are in agreement with previous reports [193,197,198,199] and strengthen the concept that IGF-1R activation is one of the mechanisms leading to the EMT and hence to EGFR-TKI resistance in LC cells. Zhou et al. also showed that the EMT is triggered by SNAIL through pERK but not through pAKT signaling, and promotes β-catenin translocation to the nucleus thereby suppressing E-cadherin (Figure 1).

Differently, Yi et al. focused on the role of the stromal microenvironment, and hence of cancer-associated fibroblasts (CAFs) in EGFR-TKI resistance and in the EMT [200]. They found that the conditioned medium from CAFs overexpresses specific factors, including IGF-1 and hepatocyte growth factor (HGF), which synergistically increase the expression and phosphorylation of annexin A2 (ANXA2), and are able to induce EMT phenotype and enhance NSCLC migratory potential (Figure 1). Moreover, inhibition of the IGF-1/IGF-1R and HGF/c-Met axes prevents both EGFR-TKI resistance and the EMT [200]. More than 50% of LC patients receive radiation therapy. However, radiation upregulates cancer-promoting genes, such as EGFR, thereby causing resistance [201]. Choi et al. demonstrated that transmembrane 4L six family member 4 (TM4SF4) protein confers radiation-resistance in lung A549 and Calu-3 adenocarcinoma cells [202]. In fact, TM4SF4 overexpression triggers the IGF-1/IGF-1R axis which in turn activates PI3K and nuclear translocation of NF-κB (Figure 1). Notably, MMP2, MMP7, and MMP9, which are biomarkers of migration and invasion ability of LC cells [203], were upregulated in TM4SF4-overexpressing NSCLC cells. This finding supports evidence that NSCLC features are EMT traits [204,205].

Likewise, hypoxia can induce the EMT in LC. Indeed, Nurwidya et al. demonstrated that IGF-1/IGF-1R/IGFBP3 are upregulated in an HIF1α-dependent manner in hypoxic A549 and HCC2935 cells [206]. By inhibiting IGF-1R signaling, the authors showed that hypoxic NSCLC cells do not develop EMT molecular hallmarks, whereas exogenous IGF-1 induces the EMT under normoxic condition [206].

### 5.3. Gastric Cancer

The invasive behavior and metastatic properties of GC are closely related to the EMT and high levels of some EMT markers in biopsy samples are hallmarks of poor prognosis [7,105,106,107,108,109,207,208]. IGF-1 signaling is the main EMT axis in GC and, in general, it follows RAS/MEK/ERK and PI3K/AKT routes, which are common to other types of cancer [105,207,208,209]. The upregulation of ZEB2, but not of ZEB1, TWIST1 or TWIST2 is the main peculiarity of IGF-1-induced EMT in GC, at least in BGC-823 human gastric adenocarcinoma cells (Figure 1) [208]. Indeed, Li et al. demonstrated that inhibition of the PI3K/AKT signaling pathway reverses ZEB2 upregulation and the subsequent EMT process mediated by IGF-1 [208]. However, they also reported that the activation of GSK-3β inhibits ZEB2 upregulation and is able to maintain the epithelial phenotype of BGC-823 [208]. It is noteworthy that ZEB2 protein levels are upregulated after IGF-1 treatment without an increase in ZEB2 mRNA, which suggests post-transcriptional regulation [105]. 

Several studies have shown that reduced levels of miR-200 family members are associated with tumor metastasis, poor disease outcome and the EMT [210,211,212,213,214]. In most cancers, this role is played particularly by miR-200b [6]. However, in some tumor setting, miR-200c seems to cause EMT suppression through ZEB1/2 targeting [211,212,213]. IGF-1 stimulation induces miR-200c downregulation in MGC-803 human gastric carcinoma cells and in SGC-7901 human metastatic gastric carcinoma cells [105]. Moreover, the PI3K/AKT inhibitor LY294002, the ERK inhibitor PD98059 and transient knockdown of ERK or AKT partially reverse the IGF-1-mediated downregulation of miR-200c [105]. Given that the IGF-1-induced EMT upregulates ZEB2 but not ZEB1, it is conceivable that miR-200c controls this program mainly by acting on ZEB2 (Figure 1). In this context, the activation of the AKT/ERK signaling, the inhibition of miR-200c expression, and the upregulation of ZEB2 could be influenced, at least indirectly, by Cbl proto-oncogene B (CBLB), whereas Cbl ubiquitin ligase maintains cell-cell adhesion and suppresses cell migration (Figure 1) [215,216,217]. In fact, IGF-1-induced EMT and migration potential are increased and miRNA-200c expression decreased in CBLB-knockdown GC cells [105]. In other cancer contexts, Cbl proto-oncogene C (CBLC) combines with IGF-1R and mediates receptor polyubiquitination in response to IGF-1 [218]. After IGF-1R phosphorylation and activation, receptor combination with CBLB initiates IGF-1R degradation in IGF-1-stimulated MGC-803 GC cells. It is noteworthy that knockdown of CBLB significantly inhibits this process [105]. Moreover, IGF-1R expression in gastric adenocarcinoma tissues is positively associated with late-stage tumors and lymph node metastasis [113], and negatively correlated with CBLB expression [105]. CBLB expression is positively associated with early-stage tumor and negatively with lymph node metastasis [105]. CBLB could repress IGF-1R and decrease the risk of developing lymph node metastasis in patients with GC [105].

Another possible crucial molecular regulator of IGF-1-mediated EMT in GC is survivin, whose expression has been associated with GC stage and metastasis (Figure 1) [207]. Indeed, IGF-1 treatment increases ERK and AKT phosphorylation in the GC cell line BGC823, and the expression of survivin and EMT biomarkers, including N-cadherin, MMP2, and SNAIL [207]. Silencing of survivin eradicates the expression of IGF-1-induced EMT biomarkers like N-cadherin, MMP2, and SNAIL, and negatively affects (reduces) the migration and invasion of BGC823 cells [207]. Moreover, interferon induces transmembrane protein 2 (IFITM2), thought to act as a tumor suppressor, promotes cancer cell proliferation, invasion, migration and the EMT in vitro, as well as tumor growth and metastasis in an xenograft model [136]. Interestingly, IGF-1 induces IFITM2 expression via IGF-1R/STAT3 signaling (Figure 1) [209]. 

In the light of these findings, strategies able to attenuate, in cancer cells, the invasive and migratory potential related to IGF-1-mediated EMT induction, have a great potential in the prevention of cancer progression and metastasis.

## 6. Conclusions

Accumulating clinical evidence shows that an overexpressed and/or hyperactivated IGF system plays an important role in the progression of many types of solid tumors, including breast, lung, and gastric cancers [37,40,41,219]. Several studies report that the enhanced activation of the IGF-1/IGF-1R signaling axis promotes cancer proliferation and survival [42,220,221]. This scenario prompted efforts to develop anticancer drugs that target the IGF system and its downstream pathways. Currently, human monoclonal blocking antibodies or TKIs are being used in preclinical studies and in clinical trials/protocols [222,223,224,225]. However, these pharmacological strategies are not producing the expected results, often due to the development of acquired resistance mechanisms in the short/medium period of treatment. This disappointing outcome can be partly explained by mutations in key genes, impaired signaling and an altered EMT profile. Indeed, many oncogenes induce an aggressive phenotype via the IGF-1-mediated EMT program. Surprisingly, the molecular and functional traits of the EMT phenotype are common to various tumors, but the molecular routes leading to such features are tumor-specific. 

Figure 1 is an overview of current knowledge about the molecular species involved in the IGF-1-mediated EMT in breast, lung, and gastric cancers. ERK/MAPK and IRS-1/PI3K/AKT/GSK-3β, the major IGF-1R signaling transduction pathways, are involved in IGF-1-mediated EMT activation. In fact, ERK/MAPK signaling pathways activate ZEB1 and TWIST, which are the main EMT transcription factors. Activation of IGF-1R signal transduction via IRS-1/PI3K/AKT/GSK-3β leads to inhibition of E-cadherin via repression of β-catenin and SNAIL by GSK-3β, thereby regulating the EMT program. Whatever the major IGF-1R signaling transduction pathway activated (PI3K/AKT and/or RAS/MEK/ERK pathway) in a human epithelial cancer, current evidence shows that there is a tumor-specific molecular signature, acting at protein and mRNA level, that enables activation of EMT-promoting transcription factors (such as SNAILs, ZEBs and TWISTs). Little is known about these signatures. Most studies focused more on the PI3K/AKT pathway than on the RAS/ERK pathway, and surprisingly, the majority report that modulators involved in EMT induction by IGF-1 act through the PI3K/AKT arm of IGF-1R downstream signaling. To our knowledge, no RAS/ERK modulators are reported in the types of cancers investigated herein. Nevertheless, both arms lead to the activation of EMT markers which, except for TWIST1, are shared by at least two cancer types reported herein.

The number of studies characterizing the IGF-1-mediated EMT activation in BC largely exceeds those devoted to other tumors. Hopefully, future studies will reveal the existence of other tumor-specific routes converging to the EMT, thus enabling our understanding of the invasive and migratory potential of cancer cells in IGF-1-mediated processes.

## Abbreviations

IGF-1	Insulin-like growth factor 1
IGF-1R	IGF-1 receptor
GH	Growth hormone
IGFBP	IGF binding protein 3
EMT	Epithelial-mesenchymal transition
ECM	Extracellular matrix
CSC	Cancer stem cell
RTK	Receptor tyrosine kinase
NCAM	Neural cell adhesion molecule
MMP	Matrix metalloproteinase
bHLH	Helix-loop-helix
ZEB	Zinc finger E-box
TGF-β	Transforming growth factor-beta
GSK-3β	Glycogen synthase kinase 3-beta
NF-κB	Nuclear factor kappa B
PI3K	Phosphoinositide 3-kinase
AKT	AKT serine/threonine kinase
HIF1α	Hypoxia-inducible factor 1α
MAPK	Mitogen-activated protein kinase
ID1	Inhibitor of DNA binding 1
mTORC	Mammalian TOR complex
ERK	Extracellular signal-regulated kinases
SHCA	SRC homology 2 domain-containing-transforming A
SOS	Son of seven-less
TNF	Tumor necrosis factor
TRAF6	TNF receptor-associated factor 6
TAK1	TGF-β-activated kinase 1
BC	Breast cancer
DDR1	Discoidin domain receptor 1
MEMO1	Mediator of ErbB2-driven cell motility 1
IRS-1	Insulin receptor substrate-1
WISP3	WNT1 inducible signaling pathway protein 3
MUC1	Mucin1
EGFR	Epidermal growth factor receptor
FAK	Focal adhesion kinase
TNBC	Triple negative breast cancer
LC	Lung cancer
NSCLC	Non-small cell lung cancer
IKBα	NF-κB inhibitor alpha
SCLC	Small cell lung cancer
TKI	Tyrosine kinase inhibitors
CAF	Cancer-associated fibroblasts
ANXA2	Annexin A2
HGF	Hepatocyte growth factor
TM4SF4	Transmembrane 4L six family member 4
GC	Gastric cancer
CBLB	Cbl proto-oncogene B
CBLC	Cbl proto-oncogene C
IFITM2	Interferon induced transmembrane protein 2
STAT3	Signal transducer and activator of transcription 3

## References

[1] Baserga R., Peruzzi F., Reiss K. (2003). The igf-1 receptor in cancer biology. Int. J. Cancer.

[2] LeRoith D., Yakar S. (2007). Mechanisms of disease: Metabolic effects of growth hormone and insulin-like growth factor 1. Nat. Clin. Pract. Endocrinol. Metab..

[3] Pollak M. (2008). Insulin and insulin-like growth factor signalling in neoplasia. Nat. Rev. Cancer.

[4] Werner H., Bruchim I. (2009). The insulin-like growth factor-i receptor as an oncogene. Arch. Physiol. Biochem..

[5] Maki R.G. (2010). Small is beautiful: Insulin-like growth factors and their role in growth, development, and cancer. J. Clin. Oncol..

[6] Lamouille S., Xu J., Derynck R. (2014). Molecular mechanisms of epithelial-mesenchymal transition. Nat. Rev. Mol. Cell Biol..

[7] Li H., Batth I.S., Qu X., Xu L., Song N., Wang R., Liu Y. (2017). Igf-ir signaling in epithelial to mesenchymal transition and targeting igf-ir therapy: Overview and new insights. Mol. Cancer.

[8] Cohen P. (2006). Overview of the igf-i system. Horm. Res..

[9] Sato Y., Inokuchi M., Takagi Y., Otsuki S., Fujimori Y., Yanaka Y., Kobayashi K., Higuchi K., Kojima K., Kawano T. (2015). Relationship between expression of igfbp7 and clinicopathological variables in gastric cancer. J. Clin. Pathol..

[10] Bach L.A., Headey S.J., Norton R.S. (2005). Igf-binding proteins—The pieces are falling into place. Trends Endocrinol. Metab..

[11] Firth S.M., Baxter R.C. (2002). Cellular actions of the insulin-like growth factor binding proteins. Endocr. Rev..

[12] Brouwer-Visser J., Huang G.S. (2015). Igf2 signaling and regulation in cancer. Cytokine Growth Factor Rev..

[13] Vishwamitra D., George S.K., Shi P., Kaseb A.O., Amin H.M. (2017). Type i insulin-like growth factor receptor signaling in hematological malignancies. Oncotarget.

[14] Laviola L., Natalicchio A., Giorgino F. (2007). The igf-i signaling pathway. Curr. Pharm. Des..

[15] Imperlini E., Mancini A., Alfieri A., Martone D., Caterino M., Orru S., Buono P. (2015). Molecular effects of supraphysiological doses of doping agents on health. Mol. Biosyst..

[16] Giustina A., Mazziotti G., Canalis E. (2008). Growth hormone, insulin-like growth factors, and the skeleton. Endocr. Rev..

[17] Pollak M. (2012). The insulin and insulin-like growth factor receptor family in neoplasia: An update. Nat. Rev. Cancer.

[18] Harrela M., Koistinen H., Kaprio J., Lehtovirta M., Tuomilehto J., Eriksson J., Tolvanen L., Koskenvuo M., Leinonen P., Koistinen R. (1996). Genetic and environmental components of interindividual variation in circulating levels of igf-i, igf-ii, igfbp-1, and igfbp-3. J. Clin. Investig..

[19] Longo V.D., Fontana L. (2010). Calorie restriction and cancer prevention: Metabolic and molecular mechanisms. Trends Pharmacol. Sci..

[20] Fontana L., Partridge L., Longo V.D. (2010). Extending healthy life span—From yeast to humans. Science.

[21] Thissen J.P., Ketelslegers J.M., Underwood L.E. (1994). Nutritional regulation of the insulin-like growth-factors. Endocr. Rev..

[22] Fontana L., Weiss E.P., Villareal D.T., Klein S., Holloszy J.O. (2008). Long-term effects of calorie or protein restriction on serum igf-1 and igfbp-3 concentration in humans. Aging Cell.

[23] Redman L.M., Veldhuis J.D., Rood J., Smith S.R., Williamson D., Ravussin E., Team P.C. (2010). The effect of caloric restriction interventions on growth hormone secretion in nonobese men and women. Aging Cell.

[24] Nishida Y., Matsubara T., Tobina T., Shindo M., Tokuyama K., Tanaka K., Tanaka H. (2010). Effect of low-intensity aerobic exercise on insulin-like growth factor-i and insulin-like growth factor-binding proteins in healthy men. Int. J. Endocrinol..

[25] Yu M., King B., Ewert E., Su X., Mardiyati N., Zhao Z., Wang W. (2016). Exercise activates p53 and negatively regulates igf-1 pathway in epidermis within a skin cancer model. PLoS ONE.

[26] Hede M.S., Salimova E., Piszczek A., Perlas E., Winn N., Nastasi T., Rosenthal N. (2012). E-peptides control bioavailability of igf-1. PLoS ONE.

[27] De Santi M., Annibalini G., Barbieri E., Villarini A., Vallorani L., Contarelli S., Berrino F., Stocchi V., Brandi G. (2016). Human igf1 pro-forms induce breast cancer cell proliferation via the igf1 receptor. Cell. Oncol..

[28] Annibalini G., Contarelli S., De Santi M., Saltarelli R., Di Patria L., Guescini M., Villarini A., Brandi G., Stocchi V., Barbieri E. (2018). The intrinsically disordered e-domains regulate the igf-1 prohormones stability, subcellular localisation and secretion. Sci. Rep..

[29] Philippou A., Maridaki M., Pneumaticos S., Koutsilieris M. (2014). The complexity of the igf1 gene splicing, posttranslational modification and bioactivity. Mol. Med..

[30] Kiess W., Paquette J., Koepf G., Wolf E., Deal C. (1999). Proinsulin-like growth factor-ii overexpression does not alter monoallelic h19 gene expression in transfected human embryonic kidney fibroblasts. Biochem. Biophys. Res. Commun..

[31] Christofori G., Naik P., Hanahan D. (1994). A second signal supplied by insulin-like growth factor ii in oncogene-induced tumorigenesis. Nature.

[32] Liu J.P., Baker J., Perkins A.S., Robertson E.J., Efstratiadis A. (1993). Mice carrying null mutations of the genes encoding insulin-like growth factor-i (igf-1) and type-1 igf receptor (igf1r). Cell.

[33] Sell C., Rubini M., Rubin R., Liu J.P., Efstratiadis A., Baserga R. (1993). Simian virus-40 large tumor-antigen is unable to transform mouse embryonic fibroblasts lacking type-1 insulin-like growth-factor receptor. Proc. Natl. Acad. Sci. USA.

[34] Bentov I., Werner H. (2004). Igf, igf receptor and overgrowth syndromes. Pediatr. Endocrinol. Rev..

[35] Werner H. (2009). For debate: The pathophysiological significance of igf-i receptor overexpression: New insights. Pediatr. Endocrinol. Rev..

[36] Werner H. (2012). Tumor suppressors govern insulin-like growth factor signaling pathways: Implications in metabolism and cancer. Oncogene.

[37] Werner H., Bruchim I. (2012). Igf-1 and brca1 signalling pathways in familial cancer. Lancet Oncol..

[38] Plymate S.R., Bae V.L., Maddison L., Quinn L.S., Ware J.L. (1997). Reexpression of the type 1 insulin-like growth factor receptor inhibits the malignant phenotype of simian virus 40 t antigen immortalized human prostate epithelial cells. Endocrinology.

[39] Damon S.E., Plymate S.R., Carroll J.M., Sprenger C.C., Dechsukhum C., Ware J.L., Roberts C.T. (2001). Transcriptional regulation of insulin-like growth factor-i receptor gene expression in prostate cancer cells. Endocrinology.

[40] Yee D., Lee A.V. (2000). Crosstalk between the insulin-like growth factors and estrogens in breast cancer. J. Mammary Gland Biol. Neoplasia.

[41] Schnarr B., Strunz K., Ohsam J., Benner A., Wacker J., Mayer D. (2000). Down-regulation of insulin-like growth factor-i receptor and insulin receptor substrate-1 expression in advanced human breast cancer. Int. J. Cancer.

[42] Samani A.A., Yakar S., LeRoith D., Brodt P. (2007). The role of the igf system in cancer growth and metastasis: Overview and recent insights. Endocr. Rev..

[43] Maor S., Mayer D., Yarden R.I., Lee A.V., Sarfstein R., Werner H., Papa M.Z. (2006). Estrogen receptor regulates insulin-like growth factor-i receptor gene expression in breast tumor cells: Involvement of transcription factor sp1. J. Endocrinol..

[44] Schayek H., Seti H., Greenberg N.M., Sun S.H., Werner H., Plymate S.R. (2010). Differential regulation of insulin-like growth factor-i receptor gene expression by wild type and mutant androgen receptor in prostate cancer cells. Mol. Cell. Endocrinol..

[45] Hellawell G.O., Turner G.D., Davies D.R., Poulsom R., Brewster S.F., Macaulay V.M. (2002). Expression of the type 1 insulin-like growth factor receptor is up-regulated in primary prostate cancer and commonly persists in metastatic disease. Cancer Res..

[46] Klammt J., Pfaffle R., Werner H., Kiess W. (2008). Igf signaling defects as causes of growth failure and iugr. Trends Endocrinol. Metab..

[47] Kruis T., Klammt J., Galli-Tsinopoulou A., Wallborn T., Schlicke M., Muller E., Kratzsch J., Korner A., Odeh R., Kiess W. (2010). Heterozygous mutation within a kinase-conserved motif of the insulin-like growth factor i receptor causes intrauterine and postnatal growth retardation. J. Clin. Endocrinol. Metab..

[48] Wallborn T., Wuller S., Klammt J., Kruis T., Kratzsch J., Schmidt G., Schlicke M., Muller E., van de Leur H.S., Kiess W. (2010). A heterozygous mutation of the insulin-like growth factor-i receptor causes retention of the nascent protein in the endoplasmic reticulum and results in intrauterine and postnatal growth retardation. J. Clin. Endocrinol. Metab..

[49] Levine A.J., Feng Z.H., Mak T.W., You H., Jin S.K. (2006). Coordination and communication between the p53 and igf-1-akt-tor signal transduction pathways. Genes Dev..

[50] Maor S.B., Abramovitch S., Erdos M.R., Brody L.C., Werner H. (2000). Brca1 suppresses insulin-like growth factor-i receptor promoter activity: Potential interaction between brca1 and sp1. Mol. Genet. Metab..

[51] Yuen J.S.P., Cockman M.E., Sullivan M., Protheroe A., Turner G.D.H., Roberts I.S., Pugh C.W., Werner H., Macaulay V.M. (2007). The vhl tumor suppressor inhibits expression of the igf1r and its loss induces igf1r upregulation in human clear cell renal carcinoma. Oncogene.

[52] Werner H., Meisel-Sharon S., Bruchim I. (2018). Oncogenic fusion proteins adopt the insulin-like growth factor signaling pathway. Mol. Cancer.

[53] Karnieli E., Werner H., Rauscher F.J., Benjamin L.E., LeRoith D. (1996). The igf-i receptor gene promoter is a molecular target for the ewing’s sarcoma wilms’ tumor 1 fusion protein. J. Biol. Chem..

[54] Werner H., Idelman G., Rubinstein M., Pattee P., Nagalla S.R., Roberts C.T. (2007). A novel ews-wt1 gene fusion product in desmoplastic small round cell tumor is a potent transactivator of the insulin-like growth factor-i receptor (igf-ir) gene. Cancer Lett..

[55] Ayalon D., Glaser T., Werner H. (2001). Transcriptional regulation of igf-i receptor gene expression by the pax3-fkhr oncoprotein. Growth Horm. IGF Res..

[56] Sharon S.M., Pozniak Y., Geiger T., Werner H. (2016). Tmprss2-erg fusion protein regulates insulin-like growth factor-1 receptor (igf1r) gene expression in prostate cancer: Involvement of transcription factor sp1. Oncotarget.

[57] Peterson J.E., Jelinek T., Kaleko M., Siddle K., Weber M.J. (1994). C-phosphorylation and activation of the igf-i receptor in src-transformed cells. J. Biol. Chem..

[58] Reiss K., Ferber A., Travali S., Porcu P., Phillips P.D., Baserga R. (1991). The protooncogene c-myb increases the expression of insulin-like growth factor-i and insulin-like growth factor-i receptor messenger-rnas by a transcriptional mechanism. Cancer Res..

[59] Travali S., Reiss K., Ferber A., Petralia S., Mercer W.E., Calabretta B., Baserga R. (1991). Constitutively expressed c-myb abrogates the requirement for insulinlike growth factor 1 in 3t3 fibroblasts. Mol. Cell. Biol..

[60] Kim S.O., Park J.G., Lee Y.I. (1996). Increased expression of the insulin-like growth factor i (igf-i) receptor gene in hepatocellular carcinoma cell lines: Implications of igf-i receptor gene activation by hepatitis b virus x gene product. Cancer Res..

[61] Jones R.A., Campbell C.I., Petrik J.J., Moorehead R.A. (2008). Characterization of a novel primary mammary tumor cell line reveals that cyclin d1 is regulated by the type i insulin-like growth factor receptor. Mol. Cancer Res..

[62] Guha N., Sonksen P.H., Holt R.I.G. (2009). Igf-i abuse in sport: Current knowledge and future prospects for detection. Growth Horm. Igf Res..

[63] Orrù S., Nigro E., Mandola A., Alfieri A., Buono P., Daniele A., Mancini A., Imperlini E. (2017). A functional interplay between igf-1 and adiponectin. Int. J. Mol. Sci..

[64] Baumann G.P. (2012). Growth hormone doping in sports: A critical review of use and detection strategies. Endocr. Rev..

[65] Hansen T.K., Gravholt C.H., Orskov H., Rasmussen M.H., Christiansen J.S., Jorgensen J.O.L. (2002). Dose dependency of the pharmacokinetics and acute lipolytic actions of growth hormone. J. Clin. Endocrinol. Metab..

[66] Chikani V., Ho K.K.Y. (2014). Action of gh on skeletal muscle function: Molecular and metabolic mechanisms. J. Mol. Endocrinol..

[67] Mancini A., Imperlini E., Alfieri A., Spaziani S., Martone D., Parisi A., Orru S., Buono P. (2013). Dht and igf-1 in peripheral blood lymphocytes: New markers for the biological passport of athletes. J. Biol. Regul. Homeost. Agents.

[68] Spaziani S., Imperlini E., Mancini A., Caterino M., Buono P., Orru S. (2014). Insulin-like growth factor 1 receptor signaling induced by supraphysiological doses of igf-1 in human peripheral blood lymphocytes. Proteomics.

[69] Imperlini E., Spaziani S., Mancini A., Caterino M., Buono P., Orru S. (2015). Synergistic effect of dht and igf-1 hyperstimulation in human peripheral blood lymphocytes. Proteomics.

[70] Sayeed A., Alam N., Trerotola M., Languino L.R. (2012). Insulin-like growth factor 1 stimulation of androgen receptor activity requires beta(1a) integrins. J. Cell. Physiol..

[71] Wu J.D., Haugk K., Woodke L., Nelson P., Coleman I., Plymate S.R. (2006). Interaction of igf signaling and the androgen receptor in prostate cancer progression. J. Cell. Biochem..

[72] Freier S., Weiss O., Eran M., Flyvbjerg A., Dahan R., Nephesh I., Safra T., Shiloni E., Raz I. (1999). Expression of the insulin-like growth factors and their receptors in adenocarcinoma of the colon. Gut.

[73] Wolpin B.M., Meyerhardt J.A., Chan A.T., Ng K., Chan J.A., Wu K., Pollak M.N., Giovannucci E.L., Fuchs C.S. (2009). Insulin, the insulin-like growth factor axis, and mortality in patients with nonmetastatic colorectal cancer. J. Clin. Oncol..

[74] Hornstein E., Shomron N. (2006). Canalization of development by micrornas. Nat. Genet..

[75] Jiang L., Liu X.Q., Chen Z.J., Jin Y., Heidbreder C.E., Kolokythas A., Wang A.X., Dai Y., Zhou X.F. (2010). Microrna-7 targets igf1r (insulin-like growth factor 1 receptor) in tongue squamous cell carcinoma cells. Biochem. J..

[76] McKinsey E.L., Parrish J.K., Irwin A.E., Niemeyer B.F., Kern H.B., Birks D.K., Jedlicka P. (2011). A novel oncogenic mechanism in ewing sarcoma involving igf pathway targeting by ews/fli1-regulated micrornas. Oncogene.

[77] Warburg O. (1956). On the origin of cancer cells. Science.

[78] Plas D.R., Thompson C.B. (2005). Akt-dependent transformation: There is more to growth than just surviving. Oncogene.

[79] Guertin D.A., Sabatini D.M. (2007). Defining the role of mtor in cancer. Cancer Cell.

[80] Farabaugh S.M., Boone D.N., Lee A.V. (2015). Role of igf1r in breast cancer subtypes, stemness, and lineage differentiation. Front. Endocrinol..

[81] Youlden D.R., Cramb S.M., Dunn N.A.M., Muller J.M., Pyke C.M., Baade P.D. (2012). The descriptive epidemiology of female breast cancer: An international comparison of screening, incidence, survival and mortality. Cancer Epidemiol..

[82] Torre L.A., Islami F., Siegel R.L., Ward E.M., Jemal A. (2017). Global cancer in women: Burden and trend. Cancer Epidemiol. Biomark. Prev..

[83] Allemani C., Matsuda T., Di Carlo V., Harewood R., Matz M., Niksic M., Bonaventure A., Valkov M., Johnson C.J., Esteve J. (2018). Global surveillance of trends in cancer survival 2000-14 (concord-3): Analysis of individual records for 37 513 025 patients diagnosed with one of 18 cancers from 322 population-based registries in 71 countries. Lancet.

[84] Zhang J., Lin Y., Sun X.J., Wang B.Y., Wang Z.H., Luo J.F., Wang L.P., Zhang S., Cao J., Tao Z.H. (2018). Biomarker assessment of the cbcsg006 trial: A randomized phase iii trial of cisplatin plus gemcitabine compared with paclitaxel plus gemcitabine as first-line therapy for patients with metastatic triple-negative breast cancer. Ann. Oncol..

[85] Abotaleb M., Kubatka P., Caprnda M., Varghese E., Zolakova B., Zubor P., Opatrilova R., Kruzliak P., Stefanicka P., Busselberg D. (2018). Chemotherapeutic agents for the treatment of metastatic breast cancer: An update. Biomed. Pharm..

[86] Ouban A., Muraca P., Yeatman T., Coppola D. (2003). Expression and distribution of insulin-like growth factor-1 receptor in human carcinomas. Hum. Pathol..

[87] Shimizu C., Hasegawa T., Tani Y., Takahashi F., Takeuchi M., Watanabe T., Ando M., Katsumata N., Fujiwara Y. (2004). Expression of insulin-like growth factor 1 receptor in primary breast cancer: Immunohistochemical analysis. Hum. Pathol..

[88] Hankinson S.E., Willett W.C., Colditz G.A., Hunter D.J., Michaud D.S., Deroo B., Rosner B., Speizer F.E., Pollak M. (1998). Circulating concentrations of insulin-like growth factor-i and risk of breast cancer. Lancet.

[89] Key T.J., Appleby P.N., Reeves G.K., Roddam A.W. (2010). Insulin-like growth factor 1 (igf1), igf binding protein 3 (igfbp3), and breast cancer risk: Pooled individual data analysis of 17 prospective studies. Lancet Oncol..

[90] Duggan C., Wang C.Y., Neuhouser M.L., Xiao L., Smith A.W., Reding K.W., Baumgartner R.N., Baumgartner K.B., Bernstein L., Ballard-Barbash R. (2013). Associations of insulin-like growth factor and insulin-like growth factor binding protein-3 with mortality in women with breast cancer. Int. J. Cancer.

[91] Jones R.A., Campbell C.I., Gunther E.J., Chodosh L.A., Petrik J.J., Khokha R., Moorehead R.A. (2007). Transgenic overexpression of igf-ir disrupts mammary ductal morphogenesis and induces tumor formation. Oncogene.

[92] Christopoulos P.F., Msaouel P., Koutsilieris M. (2015). The role of the insulin-like growth factor-1 system in breast cancer. Mol. Cancer.

[93] Motallebnezhad M., Aghebati-Maleki L., Jadidi-Niaragh F., Nickho H., Samadi-Kafil H., Shamsasenjan K., Yousefi M. (2016). The insulin-like growth factor-i receptor (igf-ir) in breast cancer: Biology and treatment strategies. Tumour Biol..

[94] Hartog H., Boezen H.M., de Jong M.M., Schaapveld M., Wesseling J., van der Graaf W.T. (2013). Prognostic value of insulin-like growth factor 1 and insulin-like growth factor binding protein 3 blood levels in breast cancer. Breast.

[95] Gradishar W.J., Yardley D.A., Layman R., Sparano J.A., Chuang E., Northfelt D.W., Schwartz G.N., Youssoufian H., Tang S., Novosiadly R. (2015). Clinical and translational results of a phase ii, randomized trial of an anti-igf-1r (cixutumumab) in women with breast cancer that progressed on endocrine therapy. Clin. Cancer Res..

[96] Kurzrock R., Patnaik A., Aisner J., Warren T., Leong S., Benjamin R., Eckhardt S.G., Eid J.E., Greig G., Habben K. (2010). A phase i study of weekly r1507, a human monoclonal antibody insulin-like growth factor-i receptor antagonist, in patients with advanced solid tumors. Clin. Cancer Res..

[97] Atzori F., Tabernero J., Cervantes A., Prudkin L., Andreu J., Rodriguez-Braun E., Domingo A., Guijarro J., Gamez C., Rodon J. (2011). A phase i pharmacokinetic and pharmacodynamic study of dalotuzumab (mk-0646), an anti-insulin-like growth factor-1 receptor monoclonal antibody, in patients with advanced solid tumors. Clin. Cancer Res..

[98] Lin E.H., Lenz H.J., Saleh M.N., Mackenzie M.J., Knost J.A., Pathiraja K., Langdon R.B., Yao S.L., Lu B.D. (2014). A randomized, phase ii study of the anti-insulin-like growth factor receptor type 1 (igf-1r) monoclonal antibody robatumumab (sch 717454) in patients with advanced colorectal cancer. Cancer Med..

[99] Cheng T.Y.D., Cramb S.M., Baade P.D., Youlden D.R., Nwogu C., Reid M.E. (2016). The international epidemiology of lung cancer: Latest trends, disparities, and tumor characteristics. J. Thorac. Oncol..

[100] Agullò-Ortuño M.T., Diaz-Garcia C.V., Agudo-Lopez A., Perez C., Cortijo A., Paz-Ares L., Lopez-Rios F., Pozo F., de Castro J., Cortes-Funes H. (2015). Relevance of insulin-like growth factor 1 receptor gene expression as a prognostic factor in non-small-cell lung cancer. J. Cancer Res. Clin. Oncol..

[101] Wang Z.G., Lu P.F., Liang Z., Zhang Z.F., Shi W.C., Cai X.B., Chen C.Y. (2015). Increased insulin-like growth factor 1 receptor (igf1r) expression in small cell lung cancer and the effect of inhibition of igf1r expression by rnai on growth of human small cell lung cancer nci-h446 cell. Growth Factors.

[102] Tas F., Bilgin E., Tastekin D., Erturk K., Duranyildiz D. (2016). Serum igf-1 and igfbp-3 levels as clinical markers for patients with lung cancer. Biomed. Rep..

[103] Izycki T., Chyczewska E., Naumnik W., Ossolinska M. (2006). Serum levels of igf-i and igfbp-3 in patients with lung cancer during chemotherapy. Oncol. Res..

[104] Wang Y.A., Sun Y., Palmer J., Solomides C., Huang L.C., Shyr Y., Dicker A.P., Lu B. (2017). Igfbp3 modulates lung tumorigenesis and cell growth through igf1 signaling. Mol. Cancer Res..

[105] Li H.M., Xu L., Li C., Zhao L., Ma Y.J., Zheng H.C., Li Z., Zhang Y., Wang R.Y., Liu Y.P. (2014). Ubiquitin ligase cbl-b represses igf-i-induced epithelial mesenchymal transition via zeb2 and microrna-200c regulation in gastric cancer cells. Mol. Cancer.

[106] Alessandrini L., Manchi M., De Re V., Dolcetti R., Canzonieri V. (2018). Proposed molecular and mirna classification of gastric cancer. Int. J. Mol. Sci..

[107] Machlowska J., Maciejewski R., Sitarz R. (2018). The pattern of signatures in gastric cancer prognosis. Int. J. Mol. Sci..

[108] Marrelli D., Polom K., Neri A., Roviello F. (2018). Clinical impact of molecular classifications in gastric cancer. Updates Surg..

[109] Li T.T., Liu H., Yu J., Shi G.Y., Zhao L.Y., Li G.X. (2018). Prognostic and predictive blood biomarkers in gastric cancer and the potential application of circulating tumor cells. World J. Gastroenterol..

[110] Orditura M., Galizia G., Sforza V., Gambardella V., Fabozzi A., Laterza M.M., Andreozzi F., Ventriglia J., Savastano B., Mabilia A. (2014). Treatment of gastric cancer. World J. Gastroenterol..

[111] Chen S., Rao H., Liu J., Geng Q., Guo J., Kong P., Li S., Liu X., Sun X., Zhan Y. (2017). Lymph nodes ratio based nomogram predicts survival of resectable gastric cancer regardless of the number of examined lymph nodes. Oncotarget.

[112] Oh S.Y., Shin A., Kim S.G., Hwang J.A., Hong S.H., Lee Y.S., Kwon H.C. (2016). Relationship between insulin-like growth factor axis gene polymorphisms and clinical outcome in advanced gastric cancer patients treated with folfox. Oncotarget.

[113] Ge J., Chen Z.K., Wu S.B., Chen J.X., Li X.L., Li J., Yin J.Y., Chen Z.H. (2009). Expression levels of insulin-like growth factor-1 and multidrug resistance-associated protein-1 indicate poor prognosis in patients with gastric cancer. Digestion.

[114] Hay E.D. (1995). An overview of epithelio-mesenchymal transformation. Acta Anat..

[115] Thiery J.P., Acloque H., Huang R.Y.J., Nieto M.A. (2009). Epithelial-mesenchymal transitions in development and disease. Cell.

[116] Thiery J.P., Sleeman J.P. (2006). Complex networks orchestrate epithelial-mesenchymal transitions. Nat. Rev. Mol. Cell Biol..

[117] Mani S.A., Guo W., Liao M.J., Eaton E.N., Ayyanan A., Zhou A.Y., Brooks M., Reinhard F., Zhang C.C., Shipitsin M. (2008). The epithelial-mesenchymal transition generates cells with properties of stem cells. Cell.

[118] Scheel C., Weinberg R.A. (2012). Cancer stem cells and epithelial-mesenchymal transition: Concepts and molecular links. Semin. Cancer Biol..

[119] Brabletz T., Jung A., Spaderna S., Hlubek F., Kirchner T. (2005). Opinion—Migrating cancer stem cells—An integrated concept of malignant tumour progression. Nat. Rev. Cancer.

[120] Jie X.X., Zhang X.Y., Xu C.J. (2017). Epithelial-to-mesenchymal transition, circulating tumor cells and cancer metastasis: Mechanisms and clinical applications. Oncotarget.

[121] Burger G.A., Danen E.H.J., Beltman J.B. (2017). Deciphering epithelial-mesenchymal transition regulatory networks in cancer through computational approaches. Front. Oncol..

[122] Yilmaz M., Christofori G. (2009). Emt, the cytoskeleton, and cancer cell invasion. Cancer Metast. Rev..

[123] Niehrs C. (2012). The complex world of wnt receptor signalling. Nat. Rev. Mol. Cell Biol..

[124] Kourtidis A., Ngok S.P., Anastasiadis P.Z. (2013). P120 catenin: An essential regulator of cadherin stability, adhesion-induced signaling, and cancer progression. Mol. Biol. Cadherins.

[125] Theveneau E., Mayor R. (2012). Cadherins in collective cell migration of mesenchymal cells. Curr. Opin. Cell Biol..

[126] Huang R.Y.J., Guilford P., Thiery J.P. (2012). Early events in cell adhesion and polarity during epithelial-mesenchymal transition. J. Cell Sci..

[127] Mendez M.G., Kojima S.I., Goldman R.D. (2010). Vimentin induces changes in cell shape, motility, and adhesion during the epithelial to mesenchymal transition. FASEB J..

[128] Yang X.F., Pursell B., Lu S.L., Chang T.K., Mercurio A.M. (2009). Regulation of beta 4-integrin expression by epigenetic modifications in the mammary gland and during the epithelial-to-mesenchymal transition. J. Cell Sci..

[129] Kim Y., Kugler M.C., Wei Y., Kim K.K., Li X.P., Brumwell A.N., Chapman H.A. (2009). Integrin alpha 3 beta 1-dependent beta-catenin phosphorylation links epithelial smad signaling to cell contacts. J. Cell Biol..

[130] Maschler S., Wirl G., Spring H., Bredow D.V., Sordat I., Beug H., Reichmann E. (2005). Tumor cell invasiveness correlates with changes in integrin expression and localization. Oncogene.

[131] Mise N., Savai R., Yu H.Y., Schwarz J., Kaminski N., Eickelberg O. (2012). Zyxin is a transforming growth factor-beta (tgf-beta)/smad3 target gene that regulates lung cancer cell motility via integrin alpha 5 beta 1. J. Biol. Chem..

[132] Koenig A., Mueller C., Hasel C., Adler G., Menke A. (2006). Collagen type i induces disruption of e-cadherin-mediated cell-cell contacts and promotes proliferation of pancreatic carcinoma cells. Cancer Res..

[133] Peinado H., Olmeda D., Cano A. (2007). Snail, zeb and bhlh factors in tumour progression: An alliance against the epithelial phenotype?. Nat. Rev. Cancer.

[134] De Craene B., Berx G. (2013). Regulatory networks defining emt during cancer initiation and progression. Nat. Rev. Cancer.

[135] Barrallo-Gimeno A., Nieto M.A. (2005). The snail genes as inducers of cell movement and survival: Implications in development and cancer. Development.

[136] Xu J., Lamouille S., Derynck R. (2009). Tgf-beta-induced epithelial to mesenchymal transition. Cell Res..

[137] Batlle E., Sancho E., Franci C., Dominguez D., Monfar M., Baulida J., de Herreros A.G. (2000). The transcription factor snail is a repressor of e-cadherin gene expression in epithelial tumour cells. Nat. Cell Biol..

[138] Cano A., Perez-Moreno M.A., Rodrigo I., Locascio A., Blanco M.J., del Barrio M.G., Portillo F., Nieto M.A. (2000). The transcription factor snail controls epithelial-mesenchymal transitions by repressing e-cadherin expression. Nat. Cell Biol..

[139] Lin T., Ponn A., Hu X., Law B.K., Lu J. (2010). Requirement of the histone demethylase lsd1 in snail-mediated transcriptional repression during epithelial-mesenchymal transition. Oncogene.

[140] Peinado H., Ballestar E., Esteller M., Cano A. (2004). Snail mediates e-cadherin repression by the recruitment of the sin3a/histone deacetylase 1 (hdac1)/hdac2 complex. Mol. Cell. Biol..

[141] Tong Z.T., Cai M.Y., Wang X.G., Kong L.L., Mai S.J., Liu Y.H., Zhang H.B., Liao Y.J., Zheng F., Zhu W. (2012). Ezh2 supports nasopharyngeal carcinoma cell aggressiveness by forming a co-repressor complex with hdac1/hdac2 and snail to inhibit e-cadherin. Oncogene.

[142] Herranz N., Pasini D., Diaz V.M., Franci C., Gutierrez A., Dave N., Escriva M., Hernandez-Munoz I., Di Croce L., Helin K. (2008). Polycomb complex 2 is required for e-cadherin repression by the snail1 transcription factor. Mol. Cell. Biol..

[143] Dong C., Wu Y., Wang Y., Wang C., Kang T., Rychahou P.G., Chi Y.I., Evers B.M., Zhou B.P. (2013). Interaction with suv39h1 is critical for snail-mediated e-cadherin repression in breast cancer. Oncogene.

[144] Dong C.F., Wu Y.D., Yao J., Wang Y.F., Yu Y.H., Rychahou P.G., Evers B.M., Zhou B.P. (2012). G9a interacts with snail and is critical for snail-mediated e-cadherin repression in human breast cancer. J. Clin. Investig..

[145] Yang M.H., Hsu D.S.S., Wang H.W., Wang H.J., Lan H.Y., Yang W.H., Huang C.H., Kao S.Y., Tzeng C.H., Tai S.K. (2010). Bmi1 is essential in twist1-induced epithelial-mesenchymal transition. Nat. Cell Biol..

[146] Hong J., Zhou J., Fu J.J., He T., Qin J., Wang L., Liao L., Xu J.M. (2011). Phosphorylation of serine 68 of twist1 by mapks stabilizes twist1 protein and promotes breast cancer cell invasiveness. Cancer Res..

[147] Postigo A.A., Depp J.L., Taylor J.J., Kroll K.L. (2003). Regulation of smad signaling through a differential recruitment of coactivators and corepressors by zeb proteins. EMBO J..

[148] Dave N., Guaita-Esteruelas S., Gutarra S., Frias A., Beltran M., Peiro S., de Herreros A.G. (2011). Functional cooperation between snail1 and twist in the regulation of zeb1 expression during epithelial to mesenchymal transition. J. Biol. Chem..

[149] Kalluri R., Weinberg R.A. (2009). The basics of epithelial-mesenchymal transition. J. Clin. Investig..

[150] Katsuno Y., Lamouille S., Derynck R. (2013). Tgf-beta signaling and epithelial-mesenchymal transition in cancer progression. Curr. Opin. Oncol..

[151] Yang Y.C., Piek E., Zavadil J., Liang D., Xie D.L., Heyer J., Pavlidis P., Kucherlapati R., Roberts A.B., Bottinger E.P. (2003). Hierarchical model of gene regulation by transforming growth factor beta. Proc. Natl. Acad. Sci. USA.

[152] Zavadil J., Bitzer M., Liang D., Yang Y.C., Massimi A., Kneitz S., Piek E., Bottinger E.P. (2001). Genetic programs of epithelial cell plasticity directed by transforming growth factor-beta. Proc. Natl. Acad. Sci. USA.

[153] Jorda M., Olmeda D., Vinyals A., Valero E., Cubillo E., Llorens A., Canoe A., Fabra A. (2005). Upregulation of mmp-9 in mdck epithelial cell line in response to expression of the snail transcription factor. J. Cell Sci..

[154] Shirakihara T., Saitoh M., Miyazono K. (2007). Differential regulation of epithelial and mesenchymal markers by delta ef1 proteins in epithelial-mesenchymal transition induced by tgf-beta. Mol. Biol. Cell.

[155] Kang Y.B., Chen C.R., Massague J. (2003). A self-enabling tgf beta response coupled to stress signaling: Smad engages stress response factor atf3 for id1 repression in epithelial cells. Mol. Cell.

[156] Nawshad A., Medici D., Liu C.C., Hay E.D. (2007). Tgf beta 3 inhibits e-cadherin gene expression in palate medial-edge epithelial cells through a smad2-smad4-lef1 transcription complex. J. Cell Sci..

[157] Kaimori A., Potter J., Kaimori J.Y., Wang C., Mezey E., Koteish A. (2007). Transforming growth factor-beta 1 induces an epithelial-to-mesenchymal transition state in mouse hepatocytes in vitro. J. Biol. Chem..

[158] Derynck R., Zhang Y.E. (2003). Smad-dependent and smad-independent pathways in tgf-beta family signalling. Nature.

[159] Moustakas A., Heldin C.H. (2005). Non-smad tgf-beta signals. J. Cell Sci..

[160] Zavadil J., Bottinger E.P. (2005). Tgf-beta and epithelial-to-mesenchymal transitions. Oncogene.

[161] Lamouille S., Derynck R. (2011). Emergence of the phosphoinositide 3-kinase-akt-mammalian target of rapamycin axis in transforming growth factor-beta-induced epithelial-mesenchymal transition. Cells Tissues Organs.

[162] Ridley A.J. (2011). Life at the leading edge. Cell.

[163] Lamouille S., Connolly E., Smyth J.W., Akhurst R.J., Derynck R. (2012). Tgf-beta-induced activation of mtor complex 2 drives epithelial-mesenchymal transition and cell invasion. J. Cell Sci..

[164] Lamouille S., Derynck R. (2007). Cell size and invasion in tgf-beta-induced epithelial to mesenchymal transition is regulated by activation of the mtor pathway. J. Cell Biol..

[165] Bakin A.V., Tomlinson A.K., Bhowmick N.A., Moses H.L., Arteaga C.L. (2000). Phosphatidylinositol 3-kinase function is required for transforming growth factor beta-mediated epithelial to mesenchymal transition and cell migration. J. Biol. Chem..

[166] Julien S., Puig I., Caretti E., Bonaventure J., Nelles L., van Roy F., Dargemont C., Garcia de Herreros A., Bellacosa A., Larue L. (2007). Activation of nf-kappa b by akt upregulates snail expression and induces epithelium mesenchyme transition. Oncogene.

[167] Lee M.K., Pardoux C., Hall M.C., Lee P.S., Warburton D., Qing J., Smith S.M., Derynck R. (2007). Tgf-beta activates erk map kinase signalling through direct phosphorylation of shca. EMBO J..

[168] Gulhati P., Bowen K.A., Liu J.Y., Stevens P.D., Rychahou P.G., Chen M., Lee E.Y., Weiss H.L., O’Connor K.L., Gao T.Y. (2011). Mtorc1 and mtorc2 regulate emt, motility, and metastasis of colorectal cancer via rhoa and rac1 signaling pathways. Cancer Res..

[169] Makrodouli E., Oikonomou E., Koc M., Andera L., Sasazuki T., Shirasawa S., Pintzas A. (2011). Braf and ras oncogenes regulate rho gtpase pathways to mediate migration and invasion properties in human colon cancer cells: A comparative study. Mol. Cancer.

[170] Kim H.J., Litzenburger B.C., Cui X., Delgado D.A., Grabiner B.C., Lin X., Lewis M.T., Gottardis M.M., Wong T.W., Attar R.M. (2007). Constitutively active type i insulin-like growth factor receptor causes transformation and xenograft growth of immortalized mammary epithelial cells and is accompanied by an epithelial-to-mesenchymal transition mediated by nf-kappab and snail. Mol. Cell. Biol..

[171] Canonici A., Steelant W., Rigot V., Khomitch-Baud A., Boutaghou-Cherid H., Bruyneel E., Van Roy F., Garrouste F., Pommier G., Andre F. (2008). Insulin-like growth factor-i receptor, e-cadherin and alpha v integrin form a dynamic complex under the control of alpha-catenin. Int. J. Cancer.

[172] Graham T.R., Zhau H.E., Odero-Marah V.A., Osunkoya A.O., Kimbro K.S., Tighiouart M., Liu T., Simons J.W., O’Regan R.M. (2008). Insulin-like growth factor-l-dependent up-regulation of zeb1 drives epithelial-to-mesenchymal transition in human prostate cancer cells. Cancer Res..

[173] Irie H.Y., Pearline R.V., Grueneberg D., Hsia M., Ravichandran P., Kothari N., Natesan S., Brugge J.S. (2005). Distinct roles of akt1 and akt2 in regulating cell migration and epithelial-mesenchymal transition. J. Cell Biol..

[174] Thiery J.P. (2002). Epithelial-mesenchymal transitions in tumour progression. Nat. Rev. Cancer.

[175] Guvakova M.A., Surmacz E. (1999). The activated insulin-like growth factor i receptor induces depolarization in breast epithelial cells characterized by actin filament disassembly and tyrosine dephosphorylation of fak, cas, and paxillin. Exp. Cell. Res..

[176] Matà R., Palladino C., Nicolosi M.L., Lo Presti A.R., Malaguarnera R., Ragusa M., Sciortino D., Morrione A., Maggiolini M., Vella V. (2016). Igf-i induces upregulation of ddr1 collagen receptor in breast cancer cells by suppressing mir-199a-5p through the pi3k/akt pathway. Oncotarget.

[177] Sorokin A.V., Chen J. (2013). Memo1, a new irs1-interacting protein, induces epithelial-mesenchymal transition in mammary epithelial cells. Oncogene.

[178] Lorenzatti G., Huang W., Pal A., Cabanillas A.M., Kleer C.G. (2011). Ccn6 (wisp3) decreases zeb1-mediated emt and invasion by attenuation of igf-1 receptor signaling in breast cancer. J. Cell Sci..

[179] Liao G., Wang M., Ou Y., Zhao Y. (2014). Igf-1-induced epithelial-mesenchymal transition in mcf-7 cells is mediated by muc1. Cell Signal.

[180] Walsh L.A., Damjanovski S. (2011). Igf-1 increases invasive potential of mcf 7 breast cancer cells and induces activation of latent tgf-beta1 resulting in epithelial to mesenchymal transition. Cell Commun. Signal.

[181] Tian M., Schiemann W.P. (2017). Tgf-beta stimulation of emt programs elicits non-genomic er-alpha activity and anti-estrogen resistance in breast cancer cells. J. Cancer Metast. Treat.

[182] Taylor M.A., Parvani J.G., Schiemann W.P. (2010). The pathophysiology of epithelial-mesenchymal transition induced by transforming growth factor-beta in normal and malignant mammary epithelial cells. J. Mammary Gland Biol. Neoplasia.

[183] Taliaferro-Smith L., Oberlick E., Liu T., McGlothen T., Alcaide T., Tobin R., Donnelly S., Commander R., Kline E., Nagaraju G.P. (2015). Fak activation is required for igf1r-mediated regulation of emt, migration, and invasion in mesenchymal triple negative breast cancer cells. Oncotarget.

[184] Nigro E., Imperlini E., Scudiero O., Monaco M.L., Polito R., Mazzarella G., Orru S., Bianco A., Daniele A. (2015). Differentially expressed and activated proteins associated with non small cell lung cancer tissues. Respir. Res..

[185] Huang L.H., Fu L.W. (2015). Mechanisms of resistance to egfr tyrosine kinase inhibitors. Acta Pharm. Sin. B.

[186] Zhou J., Wang J.J., Zeng Y.Y., Zhang X., Hu Q.T., Zheng J.H., Chen B., Xie B., Zhang W.M. (2015). Implication of epithelial-mesenchymal transition in igf1r-induced resistance to egfr-tkis in advanced non-small cell lung cancer. Oncotarget.

[187] Shih J.Y., Gow C.H., Yang P.C. (2005). Egfr mutation conferring primary resistance to gefitinib in non-small-cell lung cancer. N. Engl. J. Med..

[188] Kobayashi S., Boggon T.J., Dayaram T., Janne P.A., Kocher O., Meyerson M., Johnson B.E., Eck M.J., Tenen D.G., Halmos B. (2005). Egfr mutation and resistance of non-small-cell lung cancer to gefitinib. N. Engl. J. Med..

[189] Bean J., Brennan C., Shih J.Y., Riely G., Viale A., Wang L., Chitale D., Motoi N., Szoke J., Broderick S. (2007). Met amplification occurs with or without t790m mutations in egfr mutant lung tumors with acquired resistance to gefitinib or erlotinib. Proc. Natl. Acad. Sci. USA.

[190] Engelman J.A., Zejnullahu K., Mitsudomi T., Song Y.C., Hyland C., Park J.O., Lindeman N., Gale C.M., Zhao X.J., Christensen J. (2007). Met amplification leads to gefitinib resistance in lung cancer by activating erbb3 signaling. Science.

[191] Sequist L.V., Waltman B.A., Dias-Santagata D., Digumarthy S., Turke A.B., Fidias P., Bergethon K., Shaw A.T., Gettinger S., Cosper A.K. (2011). Genotypic and histological evolution of lung cancers acquiring resistance to egfr inhibitors. Sci. Transl. Med..

[192] Ohashi K., Sequist L.V., Arcila M.E., Moran T., Chmielecki J., Lin Y.L., Pan Y.M., Wang L., de Stanchina E., Shien K. (2012). Lung cancers with acquired resistance to egfr inhibitors occasionally harbor braf gene mutations but lack mutations in kras, nras, or mek1. Proc. Natl. Acad. Sci. USA.

[193] Guix M., Faber A.C., Wang S.E., Olivares M.G., Song Y., Qu S., Rinehart C., Seidel B., Yee D., Arteaga C.L. (2008). Acquired resistance to egfr tyrosine kinase inhibitors in cancer cells is mediated by loss of igf-binding proteins. J. Clin. Investig..

[194] Ware K.E., Marshall M.E., Heasley L.R., Marek L., Hinz T.K., Hercule P., Helfrich B.A., Doebele R.C., Heasley L.E. (2010). Rapidly acquired resistance to egfr tyrosine kinase inhibitors in nsclc cell lines through de-repression of fgfr2 and fgfr3 expression. PLoS ONE.

[195] Shien K., Yamamoto H., Soh J., Miyoshi S., Toyooka S. (2014). Drug resistance to egfr tyrosine kinase inhibitors for non-small cell lung cancer. Acta Med. Okayama.

[196] Byers L.A., Diao L.X., Wang J., Saintigny P., Girard L., Peyton M., Shen L., Fan Y.H., Giri U., Tumula P.K. (2013). An epithelial-mesenchymal transition gene signature predicts resistance to egfr and pi3k inhibitors and identifies axl as a therapeutic target for overcoming egfr inhibitor resistance. Clin. Cancer Res..

[197] Morgillo F., Woo J.K., Kim E.S., Hong W.K., Lee H.Y. (2006). Heterodimerization of insulin-like growth factor receptor/epidermal growth factor receptor and induction of survivin expression counteract the antitumor action of erlotinib. Cancer Res..

[198] Vazquez-Martin A., Cufi S., Oliveras-Ferraros C., Torres-Garcia V.Z., Corominas-Faja B., Cuyas E., Bonavia R., Visa J., Martin-Castillo B., Barrajon-Catalan E. (2013). Igf-1r/epithelial-to-mesenchymal transition (emt) crosstalk suppresses the erlotinib-sensitizing effect of egfr exon 19 deletion mutations. Sci. Rep..

[199] Cortot A.B., Repellin C.E., Shimamura T., Capelletti M., Zejnullahu K., Ercan D., Christensen J.G., Wong K.K., Gray N.S., Janne P.A. (2013). Resistance to irreversible egf receptor tyrosine kinase inhibitors through a multistep mechanism involving the igf1r pathway. Cancer Res..

[200] Yi Y.M., Zeng S.S., Wang Z.T., Wu M.H., Ma Y.H., Ye X.X., Zhang B., Liu H. (2018). Cancer-associated fibroblasts promote epithelial-mesenchymal transition and egfr-tki resistance of non-small cell lung cancers via hgf/igf-1/anxa2 signaling. Biochim. Biophys. Acta Mol. Basis Dis..

[201] Selzer E., Kornek G. (2013). Targeted drugs in combination with radiotherapy for the treatment of solid tumors: Current state and future developments. Expert Rev. Clin. Pharmacol..

[202] Choi S.I., Kim S.Y., Lee J., Cho E.W., Kim I.G. (2014). Tm4sf4 overexpression in radiation-resistant lung carcinoma cells activates igf1r via elevation of igf1. Oncotarget.

[203] Merchant N., Nagaraju G.P., Rajitha B., Lammata S., Jella K.K., Buchwald Z.S., Lakka S.S., Ali A.N. (2017). Matrix metalloproteinases: Their functional role in lung cancer. Carcinogenesis.

[204] Iwatsuki M., Mimori K., Yokobori T., Ishi H., Beppu T., Nakamori S., Baba H., Mori M. (2010). Epithelial-mesenchymal transition in cancer development and its clinical significance. Cancer Sci..

[205] Wan L.L., Pantel K., Kang Y.B. (2013). Tumor metastasis: Moving new biological insights into the clinic. Nat. Med..

[206] Nurwidya F., Takahashi F., Kobayashi I., Murakami A., Kato M., Minakata K., Nara T., Hashimoto M., Yagishita S., Baskoro H. (2014). Treatment with insulin-like growth factor 1 receptor inhibitor reverses hypoxia-induced epithelial-mesenchymal transition in non-small cell lung cancer. Biochem. Biophys. Res. Commun..

[207] Li C.J., Li J.B., Wu D.W., Han G. (2016). The involvement of survivin in insulin-like growth factor 1-induced epithelial-mesenchymal transition in gastric cancer. Tumor Biol..

[208] Li H.M., Xu L., Zhao L., Ma Y.J., Zhu Z.T., Liu Y.P., Qu X.J. (2015). Insulin-like growth factor-i induces epithelial to mesenchymal transition via gsk-3 beta and zeb2 in the bgc-823 gastric cancer cell line. Oncol. Lett..

[209] Xu L., Zhou R., Yuan L.Z., Wang S.Q., Li X.Y., Ma H.R., Zhou M.Y., Pan C.Q., Zhang J.W., Huang N. (2017). Igf1/igf1r/stat3 signaling-inducible ifitm2 promotes gastric cancer growth and metastasis. Cancer Lett..

[210] Yu J., Ohuchida K., Mizumoto K., Sato N., Kayashima T., Fujita H., Nakata K., Tanaka M. (2010). Microrna, hsa-mir-200c, is an independent prognostic factor in pancreatic cancer and its upregulation inhibits pancreatic cancer invasion but increases cell proliferation. Mol. Cancer.

[211] Romero-Pérez L., Lopez-Garcia M.A., Diaz-Martin J., Biscuola M., Castilla M.A., Tafe L.J., Garg K., Oliva E., Matias-Guiu X., Soslow R.A. (2013). Zeb1 overexpression associated with e-cadherin and microrna-200 downregulation is characteristic of undifferentiated endometrial carcinoma. Mod. Pathol..

[212] Bracken C.P., Gregory P.A., Kolesnikoff N., Bert A.G., Wang J., Shannon M.F., Goodall G.J. (2008). A double-negative feedback loop between zeb1-sip1 and the microrna-200 family regulates epithelial-mesenchymal transition. Cancer Res..

[213] Korpal M., Lee E.S., Hu G.H., Kang Y.B. (2008). The mir-200 family inhibits epithelial-mesenchymal transition and cancer cell migration by direct targeting of e-cadherin transcriptional repressors zeb1 and zeb2. J. Biol. Chem..

[214] Wang X.G., Chen X.Y., Wang R., Xiao P., Xu Z.H., Chen L., Hang W.W., Ruan A.M., Yang H.M., Zhang X.P. (2013). Microrna-200c modulates the epithelial-to-mesenchymal transition in human renal cell carcinoma metastasis. Oncol. Rep..

[215] Duan L., Raja S.M., Chen G.S., Virmani S., Williams S.H., Clubb R.J., Mukhopadhyay C., Rainey M.A., Ying G.G., Dimri M. (2011). Negative regulation of egfr-vav2 signaling axis by cbl ubiquitin ligase controls egf receptor-mediated epithelial cell adherens junction dynamics and cell migration. J. Biol. Chem..

[216] Qu X.J., Liu Y.P., Ma Y.J., Zhang Y., Li Y.C., Hou K.Z. (2008). Up-regulation of the cbl family of ubiquitin ligases is involved in atra and bufalin-induced cell adhesion but not cell differentiation. Biochem. Biophys. Res. Commun..

[217] Fournier T.M., Lamorte L., Maroun C.R., Lupher M., Band H., Langdon W., Park M. (2000). Cbl-transforming variants trigger a cascade of molecular alterations that lead to epithelial mesenchymal conversion. Mol. Biol. Cell.

[218] Sehat B., Andersson S., Girnita L., Larsson O. (2008). Identification of c-cbl as a new ligase for insulin-like growth factor-i receptor with distinct roles from mdm2 in receptor ubiquitination and endocytosis. Cancer Res..

[219] Numata K., Oshima T., Sakamaki K., Yoshihara K., Aoyama T., Hayashi T., Yamada T., Sato T., Cho H., Shiozawa M. (2016). Clinical significance of igf1r gene expression in patients with stage ii/iii gastric cancer who receive curative surgery and adjuvant chemotherapy with s-1. J. Cancer Res. Clin. Oncol..

[220] Wang Y.H., Han X.D., Qiu Y., Xiong J., Yu Y., Wang B., Zhu Z.Z., Qian B.P., Chen Y.X., Wang S.F. (2012). Increased expression of insulin-like growth factor-1 receptor is correlated with tumor metastasis and prognosis in patients with osteosarcoma. J. Surg. Oncol..

[221] Denduluri S.K., Idowu O., Wang Z.L., Liao Z., Yan Z.J., Mohammed M.K., Ye J.X., Wei Q., Wang J., Zhao L.G. (2015). Insulin-like growth factor (igf) signaling in tumorigenesis and the development of cancer drug resistance. Genes Dis..

[222] Rodon J., DeSantos V., Ferry R.J., Kurzrock R. (2008). Early drug development of inhibitors of the insulin-like growth factor-i receptor pathway: Lessons from the first clinical trials. Mol. Cancer Ther..

[223] Malaguarnera R., Belfiore A. (2011). The insulin receptor: A new target for cancer therapy. Front. Endocrinol..

[224] Yap T.A., Olmos D., Molife L.R., de Bono J.S. (2011). Targeting the insulin-like growth factor signaling pathway: Figitumumab and other novel anticancer strategies. Expert Opin. Investig. Drugs.

[225] Guha M. (2013). Anticancer igf1r classes take more knocks. Nat. Rev. Drug. Dis..

